# CRISPR-Cas Systems in Gut Microbiome of Children with Autism Spectrum Disorders

**DOI:** 10.3390/life12030367

**Published:** 2022-03-03

**Authors:** Natalia V. Zakharevich, Mikhail S. Nikitin, Alexey S. Kovtun, Vsevolod O. Malov, Olga V. Averina, Valery N. Danilenko, Irena I. Artamonova

**Affiliations:** 1 Vavilov Institute of General Genetics, Russian Academy of Sciences (RAS), 119333 Moscow, Russia; zakharevich@yandex.ru (N.V.Z.); mikhail.nikitin@phystech.edu (M.S.N.); a.kovtun@skoltech.ru (A.S.K.); olgavr06@mail.ru (O.V.A.); valerid@vigg.ru (V.N.D.); 2 Moscow Institute of Physics and Technology, State University, 141701 Dolgoprudny, Russia; 3 Skolkovo Institute of Science and Technology, 121205 Moscow, Russia; 4 Faculty of Biology, Lomonosov Moscow State University, 119991 Moscow, Russia; malov.vsevolod@yandex.ru; 5 Kharkevich Institute for Information Transmission Problems, Russian Academy of Sciences (RAS), 127051 Moscow, Russia

**Keywords:** microbiome, autism spectrum disorders, CRISPR-Cas, protospacer

## Abstract

The human gut microbiome is associated with various diseases, including autism spectrum disorders (ASD). Variations of the taxonomical composition in the gut microbiome of children with ASD have been observed repeatedly. However, features and parameters of the microbiome CRISPR-Cas systems in ASD have not been investigated yet. Here, we demonstrate such an analysis in order to describe the overall changes in the microbiome CRISPR-Cas systems during ASD as well as to reveal their potential to be used in diagnostics and therapy. For the systems identification, we used a combination of the publicly available tools suited for completed genomes with subsequent filtrations. In the considered data, the microbiomes of children with ASD contained fewer arrays per Gb of assembly than the control group, but the arrays included more spacers on average. CRISPR arrays from the microbiomes of children with ASD differed from the control group neither in the fractions of spacers with protospacers from known genomes, nor in the sets of known bacteriophages providing protospacers. Almost all bacterial protospacers of the gut microbiome systems for both children with ASD and the healthy ones were located in prophage islands, leaving no room for the systems to participate in the interspecies competition.

## 1. Introduction

Among all possible natural communities suitable for metagenomics studies, the human microbiome deserves special attention due to its medical importance. It is becoming clear that microbes populating the human body are involved in different processes important for the host and closely linked to its health. Among them are so sophisticated ones as the formation of the host’s immune system [1] and regulation of metabolic processes [2]. Gut microbiota also can affect neurological functions and even the behavior of the host [3]. Thus, the emergence of more and more evidence for the microbiome association with various diseases comes as no surprise.

For quite a long time, the human microbiome has been unattainable for complex experimental studies as a considerable fraction of bacterial species is uncultivated. According to the recent estimations, in metagenomes this fraction makes up as much as 72% of bacterial and 69% of archaeal species [4]. Only forty years ago, new methods allowed sequencing of the genes encoding 5S [5] and 16S RNAs [6], which became a new milestone in the taxonomic analysis of the microbiome [7]. For the whole genomes of organisms shaping the human microbiome, the problem was solved in principle only with the introduction of the next-generation sequencing technologies, when the direct sequencing of natural environmental samples had become possible. Massive sequencing of the human microbiome was targeted by several international projects, such as the Human Microbiome Project [8] and MetaHIT [9], and a series of national ones [10,11,12]. It was shown that the taxonomic composition of the sequenced samples varied dramatically among different parts of the human body they originated from and was changing gradually among them. Due to that and based on the simplicity of obtaining probes, it became customary to discuss microbiomes of separate human organs or cavities. For instance, within the HMP projects, several probes from each donor were sequenced—namely nasal, oral, vaginal, gut and skin microbiomes. Gut (or intestinal) microbiome turned out to be highly diverse and became the most popular one for investigations.

Development of the next-generation sequencing technologies and the metagenomic approach made it possible not only to describe the taxonomic composition of the studied microbiomes but also to evaluate the impact of pathogens on human health and to study complex changes in the entire microbial community associated with specific diseases. For example, for the gut microbiome, a complex relationship between taxonomic distribution and a variety of diseases was demonstrated. Among them were Crohn’s disease [13], Ulcerative colitis [14], obesity [15] and others. In addition, further research revealed a complex relationship between the gut microbiome and the brain. Now, this two-way relationship is usually called the microbiome–gut–brain axis, and it is being actively studied [16]. Recent research in this area shows a link between neurological diseases, such as Alzheimer’s [17] and Parkinson’s [18] diseases, and autism spectrum disorder (ASD) [19].

The first evidence for the relationship between the gut microbiome and ASD appeared at the turn of the millennium, when the development of regressive ASD as a response to the antibiotic treatment for chronic otitis had been documented [20]. It was suggested that Clostridiales bacteria might be associated with ASD due to the neurotoxin production [21]. Later this suggestion was supported by several studies, including a clinical trial on minocycline effects in patients with autism [22] and an investigation of alterations in the upper and lower intestinal flora of children with late-onset autism [23]. A significant step for the treatment of autism symptoms was provided by the study [24], which demonstrated the behavioral improvements for ASD children treated with Clostridiales-targeting antibiotics. Finally, it was shown that stool samples from children with ASD contained different types of Clostridiales compared to neurotypical patients [25], with *Clostridium (Lachnoclostridium) bolteae* being typical for children with ASD and gastrointestinal disorders [26]. In addition to *Clostridium*, various works also described such marker genera of autism as *Nitriliruptor*, *Youngiibacter*, *Burkholderia*, *Bilophila*, *Constrictibacter*, *Dichelobacter*, *Bacteroides* and *Prevotella*, as well as two orders—Desulfovibrionales and Methanomicrobiales [27,28].

However, some observations of the changes in the gut microbiota of patients with ASD may look contradictory. For example, there is evidence that the composition of ASD patients’ microbiome differed significantly in comparison with the healthy ones, showing lower abundances of *Bifidobacterium* species and higher abundances of *Lactobacillus* species [29], or lower abundances of the genera *Prevotella*, *Coprococcus*, and unclassified Veillonellaceae in autistic samples [30]. Additionally, several other studies demonstrated reduced overall richness of microbiome in ASD patients compared to the neurotypical group [31,32]. On the other hand, there were studies, where no significant difference [33], or even higher richness of ASD patients gut microbiome had been observed [34]. Surprisingly, nothing was reported about the role of bacteriophages in the process of the microbiome changes in the course of ASD.

Namely, the described inconsistencies in the compositional changes of the gut microbiome in ASD inspired our interest in the CRISPR-Cas systems distribution and peculiarities in ASD. Furthermore, the publicly available data resulted from the whole genome sequencing of the microbiome probes from the children with ASD have emerged recently and became a reason for this study [35]. The original experimental study was organised in three independent series of collecting different, non-numerous and non-equal, groups of patients and controls and sequencing using different protocols. The analysis of such data is further complicated by the non-balanced content of males and females and slightly different ranges of age in both target and control groups. However, obtaining the first results and formation of the general hypotheses can be possible in such analysis.

CRISPR-Cas systems (Clustered Regularly Interspaced Short Palindromic Repeats and CRISPR-associated proteins) are coded in special loci of bacteria and archaea. CRISPR arrays combine tandemly repeated short fragments interspersed with commensurate sequences unique for the genome. The latter is called ‘spacers’ and often demonstrate similarity to genetic mobile elements such as phages or plasmids. This feature reflects the spacers’ origin and provides the systems with their main function to defend against invading nucleic acids [36]. The unit of a new spacer and an additional copy of the direct repeat is inserted between the leader sequence (a genome segment separating the array from the *cas* genes) and the array, if the host cell has been able to repel an attack of an invader. Thus, the array represents a unique record of the last infections of the particular cell or its vertical descendants in chronological order [37].

Whereas cultivated bacteria served as a main object for investigations of CRISPR-Cas systems, metagenomics data had recently begun to be in demand in this area. Several studies were conducted from the description of systems in different ecological niches (e.g., [38]) for a targeted search for new systems with editing potential (e.g., [39]). The human microbiome was not set aside here as well, being studied with different approaches for arrays’ identification and analysis (e.g., [40,41]). In all these studies, an emphasis was made on the natural CRISPR-Cas systems in healthy human microbiomes.

In this study, we compared the CRISPR-Cas systems in the gut microbiomes of children with ASD and the control group in order to trace disease-mediated changes in their arrays, if any. The deeper analysis of protospacers became the second goal of this study because it could not be ruled out that CRISPR arrays contain preferably traces of encounters with specific groups of viruses during ASD. Finally, as almost all previous research had focused on the inhabitants of a healthy microbiota, the possibility of discovering previously unknown CRISPR-Cas systems in a disease-modified human microbiome also seemed promising.

## 2. Data and Algorithms

The raw data used for the study were downloaded from the NCBI BioProject collection (PRJNA516054). These data were initially obtained by sequencing of faecal probes from 77 children aged 1 to 9 years old, 54 of which were diagnosed with autism spectrum disorders according to DSM-V criteria (Diagnostic and Statistical Manual of mental disorders, fifth edition) [35]. In the original study sequencing was carried out in three independent series of experiments with different protocols on different platforms, which is illustrated in Table 1. No other differences in the parameters of the sample preparation and sequencing among series were indicated in the original manuscript [35]. The downloaded data were processed and assembled as described in [35]. Contigs with a length of less than 200 nt were dropped.

**Figure 1:**
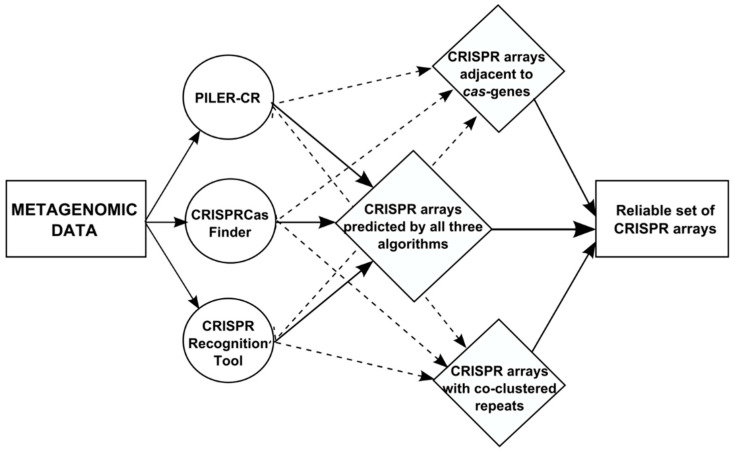
Schematic illustration of the algorithm for identification of reliable CRISPR arrays (see [38]).

For identification of CRISPR arrays, we started from the procedure described in [38] and modified it by changing CRISPRFinder to CRISPRCasFinder [42]. Its aim was to reduce false positive results produced by any of the tools used here. For that, only arrays predicted with all algorithms simultaneously or supported by one of the known biological properties of the real arrays, such as co-localisation with *cas* genes or similarity of the repeat sequence with other direct repeats, were selected for the analysis. For clustering of direct repeats for the arrays found by any of three tools we used the DNACLUST software [43] with a similarity threshold of 0.8. The resulted algorithm is illustrated in Figure 1. All procedures were performed for different series separately.

In order to test whether differences in parameters of CRISPR-Cas systems and their distribution were significant between microbiomes of children with ASD and the control group, we compared the observation values for the parameters between datasets of ASD and the control for each series. For that we used the Shapiro–Wilk test for normality and Welch’s *t*-test for equality of expectations implemented in the Python scipy.stats package. For single comparisons, the significance level of 0.05 was selected. As the test for normality may provide unreliable results for small samples, we also performed the Mann–Whitney–Wilcoxon nonparametric test to confirm the results obtained with Welch’s *t*-test. It was performed with the same significance level using its implementation in the Python scipy.stats package to confirm the results obtained with Welch’s *t*-test.

The arrays were treated as complete if they were flanked by at least 200 nucleotides on both sides in their contigs. To test the possibility of combining the data of the datasets, we compared the respective values for ASD or the control for different series using Welch’s *t*-test and confirmed the conclusions with the Mann–Whitney–Wilcoxon test.

To search for the disease markers among direct repeats, we selected clusters containing repeats from individual samples of children with ASD and not from the control group. Only a precise identity of sequences was allowed in the similar procedure for spacers.

The CRISPR-Cas systems of *Enterocloster bolteae* were identified using CRISPRCasdb [44] and its utilities in two known strains of the species. To investigate whether these systems might be used as the disease markers, we searched for the respective repeats in the microbiome data using the BLAST package [45].

To check whether microbiome strains of *Enterocloster bolteae* could contain CRISPR-Cas systems with other repeats, we annotated all metagenomic contigs with MMseqs2 [46] and selected those assigned to the species. The results of the general procedure of arrays identification for these contigs were analysed in addition to independent results of CRISPRCasFinder [42].

To find protospacers among bacteria and phages, we aligned spacer sequences to the bacterial and viral sections of the RefSeq database using BLAST [47]. To exclude hits to CRISPR arrays in bacteria, we also aligned the sequences of the respective direct repeats with the same procedure. If a spacer and the corresponding repeat had been aligned to the same bacterium, such case was excluded from the consideration. For each remaining hit, the alignment was extended up to the full coverage of the spacer using the in-house python script. After that, for the final set of protospacers, we selected hits with no more than four mismatches only.

We also analysed the locations of the protospacers in known bacterial genomes. For that purpose, we considered assemblies of bacterial genomes from the genomic subsection of the RefSeq database. If an assembly contained protospacers for arrays from more than ten metagenomic samples from any of the datasets, it was selected for further consideration. For the selected assemblies, we identified regions of potentially phage origin with two tools—Prophage Hunter [48] and PHAST [49], with default parameters for both. If the location of a protospacer had not been included into such a region, we analysed the neighbouring gene(s) using their annotation and/or best blast hits of their products. The location was marked as «prophage», if it had been included in a prophage region by at least one of the tools, or the annotation of the respective protein(s), or any of its (their) best blast hits indicated by the phage origin.

## 3. Results

### 3.1. Distribution of the CRISPR-Cas Loci and Its Parameters

For our analysis we used publicly available sequencing data of the microbiomes for children with ASD and the control group. In the original study, faeces samples of 54 children with ASD and 23 healthy children aged 1 to 9 years old were sequenced in three independent series, differing in the sequencing protocols (Table 1) [35]. After appropriate processing of the sequencing data, we performed the prediction of CRISPR arrays using the procedure developed in [38] with CRISPRFinder replaced by its improved version CRISPRCasFinder [42]. The improvement of the procedure allowed us to predict not only the reliable list of CRISPR arrays but also cas genes located nearby, where possible (Figure 1).

**Figure 2:**
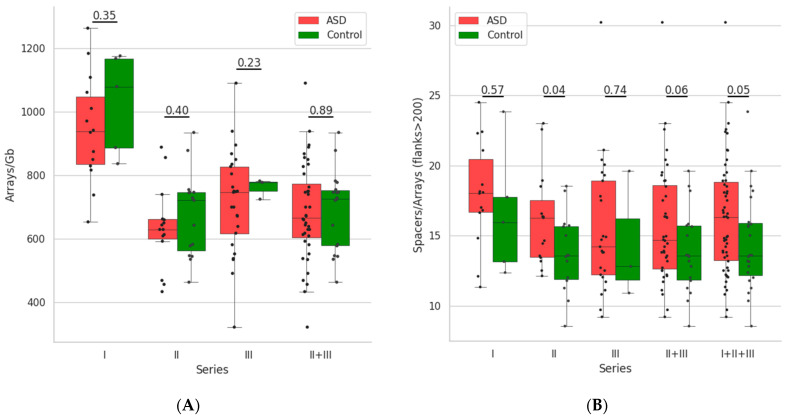
Box plots of the arrays’ numbers per Gb of assembly (**A**) and numbers of spacers per array (**B**) and the *p*-values of their comparisons for ASD and the control in all series and their allowed combinations (see Text). *p*-values are indicated above pairs of box plots for the compared datasets.

The number of the arrays normalised to the size of the microbiome assembly varies from 321.43 to 1264.29 arrays per Gb for individual metagenomes. Parameters of the array distribution for individual samples are illustrated in Supplementary Table S1, Figure 2 and Table 2.

In order to compare the occurrence of CRISPR-Cas systems in individual microbiomes between children with ASD and the control group, we performed statistical analysis independently for each series. The reason for that is the sensitivity of the number of identified CRISPR arrays to sequencing and assembly parameters, particularly read length. Namely, these parameters varied among the protocols for the data obtained in the different series (Table 1). Thus, we could not analyse all the data simultaneously straightaway. Instead, we had to check the coincidence of the distribution expectations first. For this purpose, we used Welsch’s adaptation of the Student’s t-test after confirmation of normality for the distributions for each dataset with the Shapiro–Wilk test (see Data and Algorithms and Figure 2).

The comparison demonstrated a smaller number of arrays in microbiomes of children with ASD, than in ones of the control group for all series. However, all the differences were insignificant even before the correction for multiple testing. To overcome this obstacle, we tried to combine the data of different series. However, the pairwise comparison of arrays’ numbers for ASD or the control in different series allowed joining only Series II and Series III, with none of them being allowed to join with Series I. However, the combined data demonstrated no significance in the test as well (Figure 2A).

Among other microbiome parameters tested for differences between children with ASD and the control group, only the length of complete arrays (i.e., average number of spacers in the arrays flanked with more than 200 nt in their contigs) demonstrated consistent results. In all comparisons, the complete arrays contained more spacers in the microbiomes of children with ASD (see Figure 2B). The differences were insignificant for all separate series except for the second one. In it, the *p*-value was 0.04, i.e., under the significance level for single comparisons. Here, according to the statistical test, combining all three series was allowed in pairs. For the combination of Series II and Series III datasets, the *p*-value was still higher than 0.05. However, after combining them with Series I datasets, it decreased to values lower than 0.05, the selected significance level for single comparisons. However, in all cases, the differences remained to be insignificant because of the need for the correction for multiple testing.

Comparisons of the other parameters for CRISPR-Cas systems’ distribution, listed in Table 2, between patients with ASD and the control group were neither significant for any dataset nor consistent among different datasets. All comparisons were also rechecked using the Mann–Whitney–Wilcoxon test with exactly the same results.

### 3.2. Search for Markers of the Disease among CRISPR-Cas Systems or Their Elements

The CRISPR-Cas systems represent a convenient platform for the method of close bacterial strains distinction [50]. That is why we considered their elements, direct repeats and particular spacers as candidates for markers of the disease. To reveal such systems and elements, we selected arrays, which shared exact or very similar repeats that were present in microbiomes of at least two children with ASD but absent in microbiomes of the control group. For each series, such sets were respectively small and not numerous. For instance, for the Series II data, including equal numbers of ASD and healthy children’s microbiomes, there were only three such sets with arrays from three different individual microbiomes each—one set with four arrays (two arrays from the same sample in the set) and two containing three arrays from different samples each. For different series, the repeats of the distinguishing sets differed substantially, and there was no repeat distinguishing children with ASD from the control group, at least in two series (data not shown).

Similarly, we failed to find particular spacers systematically distinguishing microbiomes of children with ASD. For example, for Series II, there were no spacers represented in more than four individual microbiomes of children with ASD and none occurred in microbiomes of the control group. Moreover, even such spacers were different for the different series.

For the opposite task, as *Clostridium bolteae* was reported as a candidate marker species of the disease because it was found in many microbiomes of the children with ASD [26], we checked our data for the presence of the CRISPR-Cas system intrinsic for the species. *C. bolteae*, recently renamed *Enterocloster bolteae* [51], is represented in the current version of CRISPRCasDB by its two strains—ATCC BAA-613 and CBBP-2. Both strains include two CRISPR loci and one *cas* locus of type IC in their genomes. Repeats of the arrays adjacent to *cas* genes differ by one nucleotide between the strains, and repeats of the distal arrays are identical. Both repeats are specific for the species, but the distal one is also present in two more species annotated as Lachnospiraceae bacterium or *Lachnoclostridium* sp. with one mismatch. We checked the presence of these two repeats in all datasets. Both repeats occurred in the microbiomes of children with ASD more frequently. The sequences identical to one of the copies of the repeat adjacent to the *cas* locus was found in 12 out of 54 individual samples of microbiomes for children with ASD in contrast to 4 out of 23 samples for the control group. The distal repeat was found in 15 samples out of 54 samples in the datasets with the ASD mark in contrast to only 2 out of 23 samples for the control group (Table 3). For the same dataset, the lists of the individual microbiome samples that contained these two repeats were different but overlapping in all cases. Thus, in our data, two CRISPR repeats of *C. boltae*, the adjacent repeat to the *cas* locus and the distal one, were present roughly one and one-third or three times more frequently in the ASD samples than in the control group, correspondingly.

In addition, we analysed metagenomic contigs annotated as *Clostridium bolteae* or *Enterocloster bolteae* with MMseqs2 in order to check whether they could contain CRISPR arrays with other direct repeats. According to automatic annotation, each individual microbiome included one or more contigs assigned to the species for both children with ASD and the control groups. However, there were no CRISPR arrays with any other direct repeat identified in the search.

### 3.3. Search for Protospacers

We searched for protospacers in the genomic assemblies from the bacterial and viral sections of the RefSeq database. To distinguish protospacers from hits with spacers in CRISPR arrays, we had also performed a parallel comparison with the array repeats (see Data and Algorithms). As a result, the protospacers were found only for a minor fraction of the spacers in these databases (Supplementary Table S1). The fraction varied from 1.7% to 31.2% and 1.9% to 36.4% for children with ASD and the control group, respectively.

We compared the fractions of spacers with protospacers in individual microbiomes between ASD and the control. According to the Welch’s t-test, we had failed to reject the null hypothesis about the equality of expectations, i.e., the distributions of the value did not differ significantly. Moreover, the mean values of the fractions did not correlate among series (Table 2 and Supplementary Table S1).

**Figure 3:**
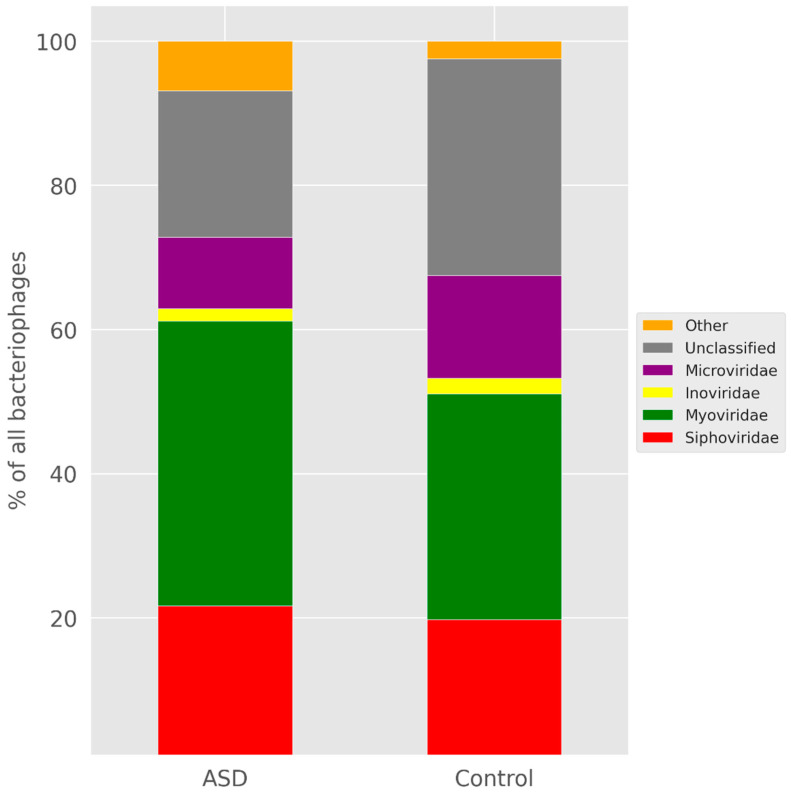
Bar plots for the relative abundance of different bacteriophage families among protospacers for the combined datasets of microbiomes for children with ASD and the control group.

We analysed the sets of bacteriophages containing protospacers. For this purpose, we compared the lists of phages providing protospacers for microbiomes of children with ASD and the control group from all our datasets. All lists were not numerous, with substantial intersections between lists for ASD and the control in each series (Supplementary Table S2). For instance, for Series II, the lists consisted of 56 phages for the case of ASD and 51 phages for the control group. The intersection of the lists of Series II included 21 organisms and, in particular, all the phages with more than four protospacers in any of the datasets (Supplementary Table S2). The comparison of the taxonomical distributions of phages for the whole combined datasets for ASD and the control did not demonstrate substantial differences in the level of families (Figure 3).

In order to analyse locations of multiple protospacers in the same known bacteria, we performed the following procedure. We selected such bacterial Refseq records that harboured protospacers for more than any ten individual microbiomes. There were no requirements on the specificity of the datasets here. For all three series, a total of 26 such records were selected by this procedure (Supplementary Table S3). Almost all protospacers in these bacteria were tightly grouped in their location. The prediction of the prophage regions with special tools and careful analysis of proteins encoded in these locations confirmed the bacteriophage origin of the corresponding chromosomal segments. Only two identified protospacers in two different bacteria had no signs of bacteriophage origin nearby (see Supplementary Table S3, “NMTQ01000037.1” and “QSFJ01000016.1” sheets). There was no preference for microbiomes of children with ASD or the control group for records either in the number of individual microbiomes obtaining hits in the analysed bacterial genomes or the total number of protospacers in each genome (data not shown).

## 4. Discussion

The problem of *a priori* CRISPR array identification has been solved quite efficiently for completed genomes [52] mainly owing to the rigid structural features of the arrays. However, the same features provide additional difficulties for sequencing assembly. That is why this problem is much more complicated for massive highly fragmented data, such as metagenomes, and is not closed yet. Computational tools designed for this purpose, such as MetaCrast [53] or CRASS [54], do not cover the area exhaustively. The former approach requires a list of predefined repeats as an input and, thus, is useless for the search of previously unknown arrays and respective systems. The latter one is strongly limited by the read length and fails to restore the order of spacers automatically in the case of respectively short reads.

An alternative approach is provided by the use of a combination of tools, suitable for completed genomes. For example, for CRISPRCasMeta, an online service for systems identification in metagenomic data [55], main modules of CRISPRCasFinder are used in combination with the CRT program. In [56], a modified version of CRT, CRT-CLI [57], was used together with Piler-CR. Here, we used a slightly updated scheme based on all three original tools, CRISPRFinder, Piler-CR and CRT, suggested in [38].

For the distribution of CRISPR arrays in microbiomes of children with ASD and the control group, the only differences we observed were in the number of arrays per Gb of assembly and the number of spacers per array (Figure 2). These differences were slight and insignificant for our datasets, yet systematic. In our opinion, the main reason for the absence of significance is the small sizes of the datasets. We failed to overcome this obstacle by combining the data of different series. The data of different series were sequenced with different protocols and demonstrated significant variance in the numbers of array per Gb of assembly, in particular, for combinations of Series I and Series II or Series I and Series III, but not in the number of spacers in arrays. The step-by-step data combining for the latter parameter was accompanied by the decrease in the *p*-value down to the value a bit lower than 0.05, the selected significance level for a single comparison. However, this observation remains insignificant after the correction for multiple comparisons.

As CRISPR-Cas systems are distributed more or less evenly among different taxa, the most probable explanation for the observed decrease in the number of arrays per Gb seems to be the general diversity reduction, which was demonstrated for the datasets we used [35]. The diversity reduction had also been observed in a number of other studies on the microbiomes of children with ASD [31,32]. However, as we already noticed in the introduction, some publications described the opposite effect [34].

Elongation of the arrays, on average, could reflect the bacteriophage burst in the ASD microbiomes, which, in turn, could be the reason for the diversity reduction. Unfortunately, almost nothing is known about the phage content of the gut microbiome in ASD. An alternative explanation for the array elongation for children with ASD could lay in the possibility of retention of the already used spacers for a bit longer due to the relaxation of the interspecies competition as a result of the diversity reduction.

All the other parameters of the CRISPR-Cas distributions did not demonstrate significant or at least consistent differences in comparisons between ASD and the control. In addition, the small sizes of the analysed datasets did not allow explaining the observed differences by any type of subsets in the analysed datasets—by males or females, by subsets of particular ages and so on.

We also tried to explain the differences in CRISPR-Cas parameters with any combination of the taxonomic units in the microbiomes using principal components analysis (data not shown), but no significant dependencies were found. The main explanation for that may lay in small fractions of raw data involved in the taxonomical representation of the individual microbiomes, as only a minority of metagenomic reads resulted from the whole genomes sequencing can be mapped onto the known genomes. Other possible explanations for that are again small sizes of the analysed datasets, heterogeneity of the disease etiologies and the respective evenness distribution of the CRISPR-Cas systems among different taxa.

There were no good candidates for markers of the disease among the elements of CRISPR-Cas systems. Neither repeats nor spacers effectively distinguished microbiomes of children with ASD from the healthy ones: there were no system elements widely spread in ASD and not occurring in the control group. All candidate elements with biased distribution were found only in a minor fraction of the ASD microbiomes and were not reproduced among the series. Thus, we believe that the bias was accidental in all these cases. The small sizes of the analysed datasets did not allow searching for systems elements occurring in both ASD and the control but significantly overrepresented in ASD. Even systems of *Enterocloster bolteae*, the only particular species named in the literature as characteristic for the ASD microbiomes, being quite specific in general, were found in ASD subjects only twice as often in the control group and were not widely spread. In our opinion, the most obvious reason for that, along with small sizes of the analysed datasets, is the heterogeneity of the disease, as the diagnosis unifies different conditions with similar symptoms but not etiology [58]. Furthermore, the dynamic changes in both the bacterial and bacteriophages compositions of the microbiome in the disease may be reflected in the instability of CRISPR-Cas systems content and, consequently, the absence of their stable widely spread elements among patients.

We expected that, according to the taxonomical changes and much more knowledge on the healthy gut microbiome, we would find notable differences in the comparison of protospacers for systems of children with ASD and the control group. That is why we compared fractions of spacers with protospacers in the known genomes and the sources of these protospacers. The formers differed neither significantly nor systematically. Furthermore, we failed to find substantial differences in the comparison of the lists of phages providing protospacers or their taxonomy. The phage lists intersected in their essential parts and all phages providing multiple protospacers belonged to the intersection. The overall number of the protospacers in phages of any family did not differ substantially between children with ASD and the control group as well. Thus, if phages do prevail in the microbiomes of children with ASD, as we suggested earlier in this section, this prevalence occurs rather due to the number of phages than to their diversity.

In the recent study on the oral microbiome, it was suggested that the CRISPR-Cas systems could participate in the interspecies competition [59] based on the comparison of the sources for protospacers of CRISPR arrays. In order to test this suggestion in the gut microbiome, both for children with ASD and the control group, we analysed the localisation of the protospacers in bacterial genomes, providing them for multiple individual samples. It was demonstrated that almost all protospacers were located in compact regions of chromosomal DNA, identified as prophage ones by special tools or based on the annotated function or best blast hits of the coded proteins. Only two protospacers, out of several hundred checked, in two different bacteria were located separately from the others and not inside or close to the genes of potentially phage origin. Therefore, the most probable function of the microbiome protospacers is the antiphage defence rather than participation in the interspecies competition. Thus, the considered phenomenon is at least not common in the gut microbiomes, both for children with ASD and for the healthy ones.

## 5. Conclusions

Here we analysed the CRISPR-Cas systems, their parameters and distribution in gut microbiota in health and disease. To the best of our knowledge, the observed differences were demonstrated for the first time. The number of samples analysed here was insufficient and the observations were not significant but systematic. That is why the described patterns need to be tested additionally on massive microbiome data. The latter may allow discovering particular contributions to the observed effects provided by the patient subsets with different sex, age and clinical traits.

Furthermore, the study provides the first indication of the bacteriophage involvement in the changes of microbiome composition in ASD. To our surprise, the bacteriophage content of the microbiomes in ASD is standing apart from the investigations in this direction. The detailed description of the taxonomical composition for the microbiome phages may shed light on the origin of the described changes in ASD.

Possibly revealing untypical functions for CRISPR-Cas systems in disease microbiomes also looks very attractive and awaits future investigation. Here, we checked the hypothesis on the involvement of CRISPR-Cas systems in the interspecies competition. It might be the small size of the analysed data that did not allow detecting such effects. That is why this needs to be tested on a more numerous dataset along with competing hypotheses.

## Figures and Tables

**Table 1:** Description of samples and statistics of sequencing and assembly important for this study (see [35]).

	Human Gut Metagenome Samples (NCBI BioProject ID: PRJNA516054)		
	Series I	Series II	Series III
Sex	both sexes		
Age (y.o.)	1–9 (ASD) 3–4 (control)	2–4 (ASD) 2–4 (control)	2–6 (ASD) 3 (control)
Number of samples	14 (ASD) 5 (control)	15 (ASD) 15 (control)	25 (ASD) 3 (control)
Platform and type of sequencing	Illumina HiSeq 2500, paired-end	Illumina HiSeq 4000, paired-end	Illumina NovaSeq 6000, paired-end
Read length, nt	135	150	150
Range of assembly size, Gb	0.06–0.26 (ASD) 0.13–0.19 (control)	0.11–0.30 (ASD) 0.14–0.28 (control)	0.11–0.37 (ASD) 0.17–0.27 (control)

**Table 2:** Parameters of individual samples, their assemblies and arrays for healthy and ASD microbiomes for all series.

Parameters		Series I	Series II	Series III
ASD	Age	4.50 ± 2.47	3.20 ± 0.77	3.60 ± 0.96
	Assembly size (Gb)	0.17 ± 0.05	0.18 ± 0.06	0.21 ± 0.07
	Arrays	161.14 ± 58.66	115.93 ± 45.23	155.08 ± 63.86
	Complete arrays (flanks > 200)	22.21 ± 10.24	22.00 ± 9.58	33.04 ± 15.34
	Arrays near *cas*	23.86 ± 9.99	25.87 ± 10.38	37.12 ± 16.69
	Spacers	1247.57 ± 488.94	895.07 ± 321.10	1241.16 ± 511.32
	Protospacers/Spacers (%)	6.09 ± 2.06	6.73 ± 2.42	4.64 ± 1.77
Control	Age	3.40 ± 0.55	2.87 ± 0.52	3.0 ± 0.0
	Assembly size (Gb)	0.16 ± 0.02	0.18 ± 0.04	0.23 ± 0.05
	Arrays	160.60 ± 16.62	122.13 ± 31.64	172.33 ± 38.53
	Complete arrays (flanks > 200)	22.00 ± 8.03	23.47 ± 5.74	37.00 ± 19.08
	Arrays near *cas*	24.80 ± 8.84	26.07 ± 5.90	39.00 ± 13.45
	Spacers	1265.40 ± 226.49	923.73 ± 277.29	1381.67 ± 465.66
	Protospacers/Spacers (%)	5.62 ± 2.17	6.36 ± 2.66	8.62 ± 2.34

**Table 3:** Direct repeats from ATCC BAA-613 and CBBP-2 strains of *Enterocloster bolteae* and numbers of individual microbiomes in which they were found.

Localisation in Relation to *cas*	ATCC BAA-613 DR	CBBP-2 DR	Series I	Series II	Series III
			ASD/ Control	ASD/ Control	ASD/ Control
Adjacent to *cas*	GTCTCCGTCCTCGCGGGCGGAGTGGGTTGAAAT	ATTTCAACCCACTCCGCCCACGAGGACGGAGAC	3/0	4/2	4/1
Distal from *cas*	ATTTCAATCCACAAGGCTCTCGCGAGCCTCGAC	GTCGAGGCTCGCGAGAGCCTTGTGGATTGAAAT	3/0	4/2	8/0

## Data Availability

Not applicable.

## Supplementary Materials

The following are available online at https://www.mdpi.com/article/10.3390/life12030367/s1, Supplementary Table S1: Parameters of CRISPR arrays for all individual samples of all series, Supplementary Table S2: Bacteriophages providing protospacers for the microbiome CRISPR-Cas systems and total number of protospacers from microbiomes of children with ASD and the control group, Supplementary Table S3: Detailed description of the 26 bacterial loci harbouring multiple protospacers. Each sheet corresponds to one bacterial RefSeq record, colouring reflects the algorithm with that the phage’s origin of the protospacer location was identified.
